# Blood Culture Testing Outcomes among Non-Malarial Febrile Children at Antimicrobial Resistance Surveillance Sites in Uganda, 2017–2018

**DOI:** 10.3390/tropicalmed6020071

**Published:** 2021-05-06

**Authors:** Rogers Kisame, Robinah Najjemba, Johan van Griensven, Freddy Eric Kitutu, Kudakwashe Takarinda, Pruthu Thekkur, Alexandre Delamou, Richard Walwema, Francis Kakooza, Ibrahim Mugerwa, Musa Sekamatte, Kimera Robert, Thomas Katairo, Marc Sam Opollo, Morgan Otita, Mohammed Lamorde

**Affiliations:** 1 Infectious Disease Institute, College of Health Sciences, Makerere University, Kampala 920102, Uganda; rwalwema@idi.co.ug (R.W.); fkakooza@idi.co.ug (F.K.); kimerarobert@gmail.com (K.R.); Motita@idi.co.ug (M.O.); mlamorde@idi.co.ug (M.L.); 2 College of Health Sciences, Makerere University School of Public Health, Kampala 920102, Uganda; 3 Public Health Consultant, 1211 Geneva, Switzerland; robinahnajjemba@yahoo.co.uk; 4 Department of Clinical Sciences, Institute of Tropical Medicine, 2000 Antwerp, Belgium; jvangriensven@itg.be; 5 Sustainable Pharmaceutical Systems (SPS) Unit, Department of Pharmacy, Makerere University School of Health Sciences, Kampala 920102, Uganda; kitutufred@gmail.com; 6 International Union against Tuberculosis and Lung Disease (The Union), 75006 Paris, France; ktakarinda@theunion.org (K.T.); pruthu.tk@theunion.org (P.T.); 7 Maferinyah Training and Research Centre, Conakry 331027, Guinea; adelamou@gmail.com; 8 Africa Centre of Excellence, Conakry 331027, Guinea; 9 Uganda National Health Laboratory Services, Kampala 920102, Uganda; mugerwaibrahim@gmail.com; 10 National One Health Platform, Ministry of Health, Kampala 920102, Uganda; musasekamatte@gmail.com; 11 Infectious Diseases Research Collaboration, Kampala 920102, Uganda; katairothomas@gmail.com; 12 Faculty of Health Sciences, Lira University, Lira P.O. Box 1035, Uganda; msopollo@gmail.com

**Keywords:** blood culture, bloodstream infections, febrile illness, antimicrobial resistance, antimicrobial susceptibility testing, operational research, SORT IT

## Abstract

Blood culture (BC) processes are critical to the utility of diagnostic testing, bloodstream infection (BSI) management, and antimicrobial resistance (AMR) surveillance. While Uganda has established BC guidelines, often laboratory practice does not meet the desired standards. This compromises pathogen recovery, reliability of antimicrobial susceptibility testing, and diagnostic test utility. This study assessed laboratory BC process outcomes among non-malarial febrile children below five years of age at five AMR surveillance sites in Uganda between 2017 and 2018. Secondary BC testing data was reviewed against established standards. Overall, 959 BC specimens were processed. Of these, 91% were from female patients, neonates, infants, and young children (1–48 months). A total of 37 AMR priority pathogens were identified; *Staphylococcus aureus* was predominant (54%), followed by *Escherichia coli* (19%). The diagnostic yield was low (4.9%). Only 6.3% of isolates were identified. AST was performed on 70% (18/26) of identified AMR priority isolates, and only 40% of these tests adhered to recommended standards. Interventions are needed to improve laboratory BC practices for effective patient management through targeted antimicrobial therapy and AMR surveillance in Uganda. Further research on process documentation, diagnostic yield, and a review of patient outcomes for all hospitalized febrile patients is needed.

## 1. Introduction

Due to multidrug resistance, bloodstream infections (BSI) are a growing public health concern and a common cause of morbidity and mortality globally [1,2,3,4]. Blood culture (BC) is a widely used laboratory procedure to diagnose BSI, guide antimicrobial therapy, and monitor AMR patterns [5]. BSIs are a major cause of morbidity and mortality among non-malarial febrile (NMF) children, especially in low and middle-income countries (LMICs) [6,7,8]. Common BC isolates reported in Africa include *Salmonella enterica* (58.4%), *Streptococcus pneumoniae* (18.3%), *Staphylococcus aureus* (9.5%), and *Escherichia coli* (7.3%) [8].

At the minimum, BC testing processes include specimen collection and transportation, culture, pathogen identification, AST, and timely report of results. Outcomes of these processes play a critical role in ensuring early and appropriate patient management. As part of a national AMR surveillance program, Uganda uses routine antibiotic sensitivity testing (AST) to inform treatment guidelines and AMR control strategies. It is intended that AMR surveillance sites adequately identify and perform AST on priority AMR pathogens (Table A1) [9]. However, there is limited laboratory capacity to perform BC and AST in accordance with recommended Clinical and Laboratory Standards Institute (CLSI) guidelines [10]. Good microbiological testing practices consist of standardized procedures for each process with a system for quality indicator monitoring to determine areas for improvements [11]. Microbiological testing and quality capacity gaps affect pathogen recovery and reliability reported for patient management and containment of AMR in Uganda. Monitoring BC processes and performance indicators such as the diagnostic yield, the proportion of specimens undergoing Gram staining, species identification, AST, and reporting of results in line with established standards plays a critical role in ensuring effective patient management and reliable AMR surveillance data [9,12,13,14,15,16,17,18].

Uganda’s Clinical Guidelines (2016) and National Action Plan for AMR (2018–2023) envision timely and reliable microbiology laboratory services, including BC, as an essential component for guiding targeted antimicrobial therapy and AMR containment. However, AMR surveillance capacity-building programs in Uganda suggest recurrent BC laboratory shortfalls [12,13]. This study assessed laboratory processes for specimen collection and transport, culture, pathogen identification, AST, reporting of results, and quality indicator monitoring of BC among NMF children under five years at five AMR surveillance sites in Uganda from October 2017 to September 2018. The study also documented the demographic and clinical characteristics of the affected under-five children, and identified the AMR pathogens and their resistance patterns.

## 2. Materials and Methods

### 2.1. Study Design

A cross-sectional study was conducted at the five national AMR surveillance sites using secondary data from routine laboratory BCs from October 2017 to September 2018.

### 2.2. Setting

#### 2.2.1. General Setting

Uganda is a low-income country in East Africa, with approximately 41.6 million people [19]. In 2020, the population of children below five years was estimated at 6.99 million with the under-five mortality rate at 64 deaths (per 1000 live births) [20]. The national health system is comprised of both private and public sectors. The country’s public health system includes a four-tiered network of hospitals located in 14 health regions managed by the Ministry of Health (MoH).

Five regional referral public hospitals with onsite microbiology laboratories were supported to initiate AMR surveillance in 2016, through provision of technical assistance to ensure the necessary infrastructure and resources were put in place. At a minimum, each AMR surveillance site has access to a network of microbiology laboratories that offer a limited range of diagnostic services including bacterial culture, pathogen identification, and AST using disk diffusion techniques. Peripheral laboratories linked to national reference laboratories (NRLs) provide specialized testing, including advanced/confirmatory pathogen identification and quantitative AST. NRLs also provide technical support to lower-level laboratories through external quality assessments, in-service training, mentorship, and laboratory supervision by subject matter experts and implementing partners.

#### 2.2.2. Specific Setting

**Figure 1:**
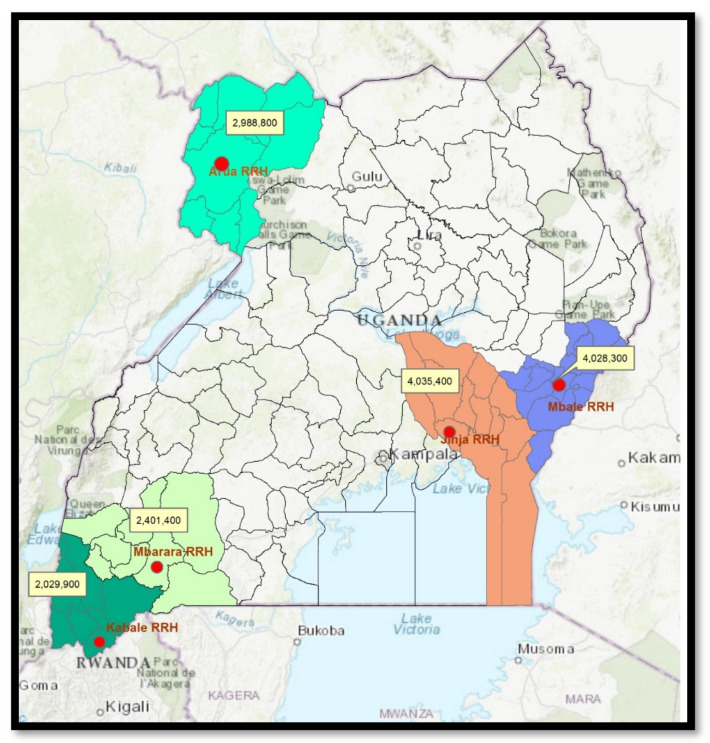
Map showing the distribution of the five AMR surveillance sites and population density for each location in Uganda. Note: The different color coding indicates separate location demonstration.

The study was conducted at the five regional referral hospitals (RRHs) in Arua, Mbale, Kabale, Jinja, and Mbarara that have participated in a national AMR surveillance program since 2016 (Figure 1). These hospitals were prioritized for diagnostic microbiology capacity building by the MoH in the early efforts to implement a structured AMR surveillance system in line with the AMR National Action Plan. These sites are supported by health sector partners such as the CDC Global Health Security Agenda program to enhance microbiology laboratory capacity for specimen collection, culture, pathogen identification, and AST [12].

The AMR surveillance program involves the use of standardized guidelines. Aggregate AST data is reported to the national AMR coordination office to inform AMR containment strategies, and subsequently to WHO via the GLASS portal. Each AMR surveillance site serves a population of at least 500,000 people in its catchment area, with approximately 50 pediatric beds and an annual caseload of 5000 febrile children, under five years of age, admitted. Each surveillance site has access to onsite BC and AST services. All surveillance site laboratories receive technical support from government and health partners to ensure AMR priority pathogens are identified, and AST is performed following CLSI guidelines [10]. Technical support includes the provision and maintenance of BC laboratory equipment, in-service training, mentorship of laboratory and clinical personnel on BC processes, and procurement of reagents and consumables. Pathogens prioritized for AMR surveillance from blood in Uganda include *Staphylococcus aureus, Escherichia coli, Klebsiella* spp., *Acinetobacter* spp., *Pseudomonas Aeruginosa*, and *Salmonella* spp.

At each surveillance site, specimens from hospitalized NMF children less than five years old were collected in BACTEC™ Plus Aerobic/F bottles and sent to the laboratory for testing. Blood culture bottles were processed onsite using the BD BACTEC™ 9050 automated system as per the manufacturer’s instructions. When bacterial growth was detected, a preliminary Gram stain was performed to determine and report a Gram-stain reaction to guide initial antimicrobial therapy. Consecutively, an inoculum was sub-cultured for species identification and AST, following CLSI guidelines. For quality assurance purposes, each positive blood culture isolate was referred to the national AMR reference laboratory for parallel testing. In addition, the designated AMR reference laboratory performed confirmation of any resistance patterns.

Laboratories were required to report preliminary results (Gram stain) and deliver all culture and AST results (pathogen identification and resistance patterns) to the clinic care unit within a turnaround time of one day from completion of testing. Samples that showed no sign of any growth after seven days were declared negative. All laboratories had a quality control system and were enrolled in an external quality assessment program administered by the reference laboratory.

Laboratory AMR surveillance data were captured in microbiology test request forms, specimen reception logbooks, and WHONET, a free Windows-based database software. Routine AST data generated at the facility level was subsequently submitted to the national AMR surveillance coordination center for validation, use, and reporting to GLASS, in support of the global actions for AMR containment.

### 2.3. Study Population

The study reviewed and analyzed secondary data from anonymized laboratory blood cultures of hospitalized, NMF children less than five years old collected from October 2017 to September 2018 at the five selected AMR surveillance sites.

### 2.4. Study Variables and Data Collection

At each AMR surveillance sentinel site, aggregate AST data were retrieved from standard WHONET files and submitted monthly to the national AMR coordination center. This data, by the BC testing process is summarized in Table 1 below:

### 2.5. Data Analysis

Routine AST data captured by surveillance sites in the WHONET software were exported and analyzed using STATA™ version 15. Categorical data were presented as frequencies and proportions in tables and figures.

## 3. Results

### 3.1. Demographic and Clinical Characteristics of Participants

A total of 959 blood culture specimens were collected from hospitalized, NMF children less than five years old at five public RRHs in the national AMR surveillance program. Jinja had the highest number of BC specimens collected, followed by Arua, Mbarara, Kabale, and Mbale, respectively (Table 2). During the study period, the majority of processed BC specimens were collected from female children (60% vs. 40% from male children, *p-*value < 0.001). Neonates, infants, and young children (1–48 months) were the main contributors of processed specimens, accounting for about 91% compared to 9% from those aged 49–59 months, *p-*value < 0.001). Patient age was not documented for three of the 959 (0.3%) specimens processed. Initial antibiotic exposure before specimen collection was recorded for only 53% of the specimens. Data on dates of fever presentation were not available.

### 3.2. Specimen Collection and Transportation

A total of 959 BC specimens were routinely collected from hospitalized, NMF children less than five years old during the study period. All of the 959 specimens did not have data on specimen volume, time of collection, and culture contamination. All laboratories did not track all prescribed quality indicators related to specimen collection and transportation.

### 3.3. Blood Culture

**Figure 2:**
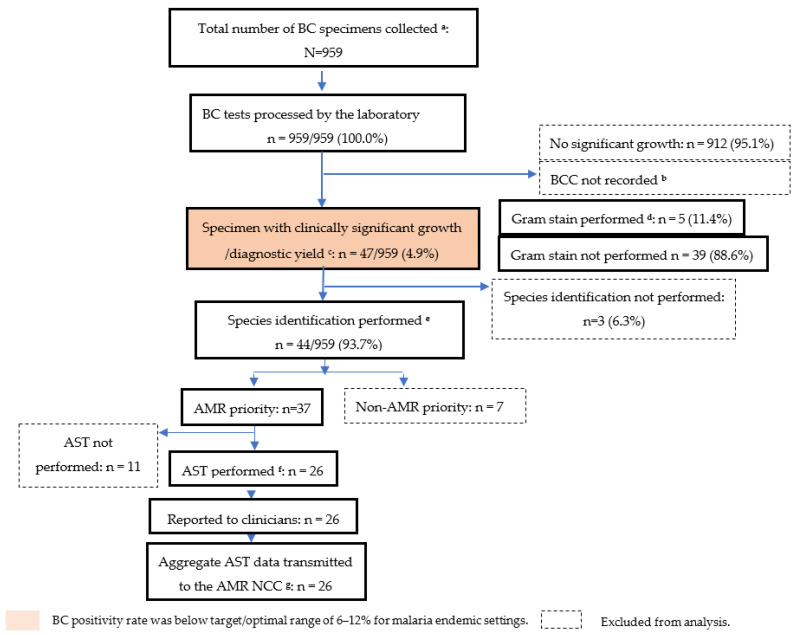
Cascade of blood culture positivity, isolate identification, and AST among hospitalized, non-malarial febrile patients undergoing blood culture at the five AMR surveillance sites in Uganda, October 2017–September 2018. ^a^ One BC specimen bottle collected from each child; ^b^ Performance target for BCC rates range from 2–3%; ^c^ Performance target for BC diagnsotic yield range from 6–12%. Only one isolate per specimen was included in our analysis; **^d^** Performance target for Gram-staining and reporting for all specimens showing clinically significant growth is 100%; ^e^ Performance target for pathogen identification and reporting is 100%; ^f^ Performance target for AST and reporting for all identified pathogens is 100%; ^g^ AST data for all AMR priority pathogens should be reported to clinicians and transmitted to the AMR NCC.

All of the 959 collected specimens underwent a complete laboratory workup to identify organisms. Of these, only 4.9% showed clinically significant growth (diagnostic yield) (Figure 2). BC contamination (BCC) was not monitored at any of the five AMR surveillance sites.

### 3.4. Pathogen Identification

Table 3 shows BC specimens collected, and the AMR priority pathogens recovered at each AMR surveillance site. Overall, 44 pathogens were recovered and identified, and no polymicrobial infections were reported (i.e., >1 pathogen recovered from one blood specimen). Of these, 37 (84%) were AMR priority pathogens. *Staphylococcus aureus* was the most predominant isolate (54%), followed by *Escherichia coli* (19%), *Salmonella* spp. (11%), *Streptococcus pneumoniae* (11%), and *Klebsiella* spp. (5%).

### 3.5. Antimicrobial Susceptibility Testing

AST was performed on only 70% of the identified AMR priority pathogens; non-AMR priority pathogens were excluded from our AST analysis. Table 4 displays resistance patterns of the five AMR priority isolates to the 14 recommended antimicrobial agents under surveillance and laboratory adherence to established AST guidelines (CLSI). Resistance to commonly used antimicrobials was observed among AMR priority pathogens. *Staphylococcus aureus*, *Escherichia coli*, *Klebsiella* spp., and *Salmonella* spp. were resistant to two or more antimicrobials. *Streptococcus pneumoniae* isolates were susceptible to all tested antimicrobial agents. Overall, only 40% of these tests adhered to recommended CLSI guidelines. Areas of non-conformity included the following:

(1) none of the recommended antimicrobial agents were tested (28%),
(2) some of the recommended antimicrobial agents were tested (22%), and
(3) incorrect pathogen–antimicrobial agent combination was used (10%).

### 3.6. Results Delivery and AMR Data Submission

During the study period, all the surveillance site laboratories did not have patient result delivery data (from the laboratory to patient care points/wards).

## 4. Discussion

This study reported findings of BC testing process outcomes, including sampled patient profiles, AMR priority pathogen etiology, quality management, and compliance to recommended AST guidelines at five RRHs, after the initiation of AMR surveillance. We identified areas for further strengthening and research. There were substantial deficits in BC laboratory process outcomes, potentially limiting blood culture test utilization in routine patient care and introducing bias regarding AMR surveillance data reported nationally.

The high number of hospitalized febrile neonates, infants, and young children (1–48 months) whose BC were processed were consistent with studies in Uganda, Switzerland, and the United States of America [10,12,21,22]. In contrast, the Switzerland study found a majority of bacterial BSI occurring in male children. These observations could be attributed to differences in the study population (children up to 15 years of age).

The low diagnostic yield/pathogen recovery (below the recommended benchmark of 6–12% considered for malaria-endemic settings). These findings are consistent with previous reports in Uganda [12]. Studies beyond Uganda reported a higher diagnostic yield [23,24]. This observation could be due to multiple factors: variations in patient sampling, inadequate specimen volumes, the number of blood specimens drawn per patient, inadequate laboratory quality management, and considerable exposure to antibiotics before sampling, as noted at the surveillance sites of Arua and Mbale that documented clinical information on antibiotic exposure before hospitalization. Antimicrobial administration before blood sample collection at the community and health facilities is common in many resource-limited settings, including Uganda [25,26,27]. Our observation of *Staphylococcus aureus* and *Escherichia coli* as the predominant AMR priority pathogens identified, and their resistance patterns were not surprising. These results were similar to prior blood culture studies [4,12,13,21,28,29]. The exception was the predominant *Salmonella* spp. isolated in Arua RRH, which likely was due to differences in underlying patient conditions, predisposing factors, specimen collection, and laboratory techniques.

Jinja RRH recovered a wider variety of AMR priority pathogens as it had the largest number of specimens processed. Arua RRH was the only surveillance site that recovered *Salmonella* spp. isolates from blood. Other factors that could influence the recovery of *Salmonella* spp. include circulating bacterial load, specimen collection timing, volumes of blood collected, and exposure to amicrobial agents before specimen collection [10,24].

Our study highlights considerable gaps in quality of laboratory performance. Most surveillance sites were unable to adhere to basic standards for culture and pathogen identification. There were variations in the identification of bacterial pathogens. The choice of antimicrobial agents for AST was not consistent with national guidelines (40% level of compliance). Laboratory performance on key quality indicators such as specimen volume, turnaround time, and specimen contamination rates was not routinely documented and monitored. Reporting of preliminary Gram-stain results for positive BC specimen was not adequately documented and tracked. Both pediatric and adult studies evaluating laboratory BC culture practices in Uganda, the Democratic Republic of Congo, Nigeria, Nepal, India, Switzerland, and the United States of America similarly reported shortfalls in microbiology laboratory quality capacity as major bottlenecks to BC execution [12,13,14,16,17,18,23,27,30]. These observations emphasize the need for targeted interventions for improving the reliability of laboratory data to guide targeted antimicrobial therapy and appropriately contribute to AMR surveillance.

Challenges related to supply chain reliability for critical laboratory commodities (including BC specimen collection supplies), personnel technical competence, and equipment functionality were potential impediments to the inadequate quality and poor compliance to standard testing guidelines. These observations are comparable to previous studies that reported laboratory supplies, personnel technical competence, and equipment functionality as major impediments to quality test utilization by clinicians [14,16,31]. Consequently, the reliability of AST reported for the national surveillance of AMR is often compromised/biased and may not sufficiently inform treatment guidelines.

To our knowledge, this study is one of the first blood culture testing cascade-analyses at AMR surveillance sites in Uganda. Strengths of our study included the inclusion of regional referral hospitals that pioneered the national AMR surveillance in 2016 and received external technical assistance and resources to enhance AST practices in support of a national AMR surveillance program. This approach minimized bias due to restrictive sampling. A systematic approach to reviewing the entire blood culture process against established technical and quality standards revealed laboratory technical challenges and their impact on patient care and AMR surveillance.

This study was limited by missing data for crucial patient demographics, clinical characteristics, and diagnostic microbiological variables. Whereas specimen collection, transportation and handling, communication of results (Gram stain, bacterial species identification, and AST), and patient outcomes are important factors in blood culture execution, this study did not assess them due to incomplete documentation and missing variables. Because of the low diagnostic yield and number of recovered pathogens (< 20 for the most predominant pathogen), this study did not perform statistical analysis comparing patient demographics and pathogens and conclude on resistance patterns to minimize potential bias. Lastly, our study focused on hospitalized non-malaria febrile children below five years of age, limiting the generalizability of pathogen etiology and their resistance patterns.

The quality of AST data generated and reported to inform treatment guidelines and AMR control strategies nationally and globally was inadequate. Site level capacity-building interventions premised on implementing laboratory procedures and practice changes could substantially improve diagnostic test utility for patient management and AMR surveillance.

## 5. Conclusions

This study has provided insight into BC testing process indicators, including specimen collection and transportation, bacterial culture, AST, and quality indicator monitoring at the five RRHs, after the initiation of AMR surveillance. Blood culture was mostly performed in neonates, infants, and young children (1–48 months). *Staphylococcus aureus*, followed by *Escherichia coli*, were the most predominant AMR priority pathogens. Laboratory BC testing process outcomes did not conform to established testing and quality standards. Interventions to improve BC testing processes, including specimen collection, bacterial culture, AST, and quality indicator monitoring at AMR surveillance sites are needed. If these processes are improved, routine microbiological diagnostic data can adequately guide targeted antimicrobial therapy for patient management, and reliably contribute to AMR surveillance in Uganda. Other studies with improved documentation of routine BC processes, a higher diagnostic yield, and analysis of patient outcomes for all hospitalized febrile patients will be necessary to further assess specimen collection practices and priority pathogen resistance patterns at AMR surveillance sites.

## Figures and Tables

**Table 1:** Summary of study data variables.

	Category of Data	Variable Description
1.	Specimen collection and transportation	Patient demographics—age, sex, and place of residence, Clinical information—date of fever presentation and antimicrobial exposure before specimen collection, Health facility name, Laboratory specimen identification number, Blood sample collection date, Specimen collection time, Staff personnel collecting the blood specimen, Specimen volume, Specimen reception date. Completion of requisition forms with all required elements (target =at least 90%)
2.	Blood culture parameters	Name of the testing laboratory, Presence of significant bacterial growth, Related quality indicators performance data—blood culture contamination (target = less than 3%) and diagnostic yield (target = 6–12%)
3.	Pathogen identification	Gram stain results, Pathogen identified (bacterial species) Gram-stain reporting (target =at least 100%) and pathogen identification (target = 100%)
4.	AST result parameters	Whether isolate was resistant, intermediate, and susceptible. AST reporting for recommended antimicrobials (target = 90%)
5.	Reporting of results and transmission parameters	Date of release of results Dates of submitting AMR data files to the national system. AST monthly data submission to the national AMR coordination unit (target = 100%).

**Table 2:** Demographic and clinical characteristics of hospitalized non-malarial febrile children undergoing blood culture at five antimicrobial resistance surveillance sites in Uganda, October 2017–September 2018.

Characteristic	Jinja n (%)	Arua n (%)	Mbarara n (%)	Kabale n (%)	Mbale n (%)	Total n (%)
Age in months ^						
<1 ^¥^	66 (8.7)	32 (27.6)	8 (25.0)	6 (20.0)	7 (36.8)	119 (12.4)
1 to 48	622 (81.6)	81 (69.8)	24 (75.0)	14 (46.7)	12 (63.2)	753 (78.5)
49–59	74 (9.7)	1 (0.9)	0 (0.0)	9 (30.0)	0 (0.0)	84 (8.8)
Not recorded	0 (0.0)	2 (1.7)	0 (0.0)	1 (3.3)	0 (0.0)	3 (0.3)
Sex ^						
Male	307 (40.3)	52 (44.8)	21 (65.6)	18 (60.0)	9 (47.4)	407 (42.4)
Female	455 (59.7)	64 (55.2)	11 (34.4)	12 (40.0)	10 (52.6)	552 (57.6)
Initial antibiotic exposure						
Yes	0 (0.0)	44 (37.9)	0 (0.0)	0 (0.0)	9 (47.4)	53 (5.5)
No *	0 (0.0)	59 (50.9)	1 (3.1)	0 (0.0)	8 (42.1)	68 (0.7)
Not recorded ^Ω^	762 (100.0)	13 (11.2)	31 (96.9)	30 (100.0)	2 (10.5)	838 (87.3)
Total N (%)	762 (79.5)	116 (12.1)	32 (3.3)	30 (3.1)	19 (2.0)	959

^ *p*-value < 0.001; ^¥^ Neonates; * Hospitalized non-malaria febrile children undergoing blood culture with a record of antibiotic exposure during the current infection at specimen collection; ^Ω^ Initial antibiotic exposure during the current infection was not recorded.

**Table 3:** Identified pathogens and yield among hospitalized/non-malarial febrile children undergoing blood culture at five antimicrobial resistance surveillance sites in Uganda, October 2017–September 2018.

Pathogen	Jinja n	Arua n	Mbarara n	Kabaale n	Mbale n	Total n
**AMR Priority Pathogens**						
**Gram-Negative**						
*Escherichia coli*	2	1	2	1	1	7
*Klebsiella* spp.	2	0	0	0	0	2
*Salmonella* spp.	0	4	0	0	0	4
**Gram-positive**						
*Staphylococcus aureus*	7	0	6	4	3	20
*Streptococcus pneumoniae*	2	0	1	1	0	4
**Non-AMR priority pathogens**						
*Staphylococcus epidermidis*	1	0	0	0	0	1
*Pseudomonas* spp.	1	0	0	0	0	1
*Candida* spp.	2	0	0	0	0	2
*Coagulase-Negative Staphylococcus*	3	0	0	0	0	3
**Diagnosic yield ^π^ (%)**	20/762 (2.6)	5/116 (4.3)	9/32 (28.1)	6/30 (20.0)	4/19 (21.0)	44/959 (4.6%)

**^π^** Diagnostic yield = total number of isolates recovered at each site/total number of blood specimens processed × 100.

**Table 4:** Compliance with antimicrobial susceptibility testing guidelines and resistance patterns of identified AMR surveillance priority pathogens among hospitalized, non-malarial febrile children undergoing blood culture at five AMR surveillance sites in Uganda, October 2017–September 2018.

Antibiotic	Gram-Negative Bacteria						Gram-Positive Bacteria			
	*E coli*, n = 3		*Klebsiella* spp., n = 1		*Salmonella* spp., n = 4		*S aureus*, n = 15		*S pneumoniae*, n = 3	
	Tested	Resistant	Tested	Resistant	Tested	Resistant	Tested	Resistant	Tested	Resistant
**AMP**	3	3	1	1	0	NR	0	NR	0	NR
**FEP**	1	0	0	NR	0	NR	1	0	0	NR
**FOX**	0	NR	0	NR	0	NR	0	NR	0	NR
**CRO**	2	1	0	NR	4	0	0	NR	0	NR
**CTX**	1	1	0	NR	0	NR	1	0	1	0
**CAZ**	1	1	0	NR	0	NR	1	1	0	NR
**CIP**	3	1	1	1	4	4	8	1	0	NR
**COL**	0	NR	0	NR	0	NR	1	0	0	NR
**GEN**	1	1	1	1	4	0	3	0	0	NR
**AMK**	2	2	0	NR	0	NR	6	0	1	0
**MEM**	0	NR	0	NR	4	0	2	2	1	0
**OXA**	0	NR	0	NR	0	NR	3	0	0	NR
**PEN**	0	NR	0	NR	0	NR	2	2	0	NR
**SXT**	0	NR	1	1	4	1	5	3	0	NR
		**Not recommended and not tested**								
		Not recommended but tested in at least one								
		Recommended and all the isolates were tested								
		Recommended but not all the isolates were tested								
		Recommended but none were tested								

Abbreviations: AMR = antimicrobial resistance; AMP = ampicillin; FEP = cefepime; FOX = cefoxitin; CRO = ceftriaxone; CTX = cefotaxime; CAZ = ceftazidime; CIP = ciprofloxacin; COL = colistin; GEN = gentamicin; AMK = amikacin; MEM = -meropenem; OXA = oxacillin; PEN = penicillin G; SXT = co-trimoxazole; NR = not reported.

## Data Availability

The data presented in this study is available on request (due to ethics and confidentiality restrictions) from the corresponding author.

## Appendix A

**Table A1:** Pathogen–antimicrobial combinations for national AMR surveillance (GLASS).

Pathogen	Antimicrobial Class	Antimicrobial Agents Used for AST ^‡^
*S. pneumoniae*	Penicillins	Penicillin G
	Sulfonamides and trimethoprim	Co-trimoxazole
	Third-generation cephalosporins	Ceftriaxone
*Staphylococcus aureus*	Penicillinase-stable beta-lactams	Cefoxitin ^b^ and Oxacillin ^a^
*Escherichia coli*	Sulfonamides and trimethoprim	Co-trimoxazole
	Fluoroquinolones	Ciprofloxacin
	Third-generation cephalosporins	Ceftriaxone and ceftazidime
	Fourth-generation cephalosporins	Cefepime
	Carbapenems	Meropenem
	Polymyxins	Colistin ^c^
	Penicillins	Ampicillin
*Klebsiella species*	Sulfonamides and trimethoprim	Co-trimoxazole
	Fluoroquinolones	Ciprofloxacin
	Third-generation cephalosporins	Ceftriaxone and ceftazidime
	Fourth-generation cephalosporins	Cefepime
	Carbapenems	Meropenem
	Polymyxins	Colistin ^c^
*Acinetobacter species*	Tetracyclines	Tigecycline
	Aminoglycosides	Gentamicin and Amikacin
	Carbapenems *	Meropenem
	Polymyxins	Colistin ^c^
*Pseudomonous Aeruginosa*	Carbapenems	Meropenem
*Salmonella* spp.	Fluoroquinolones	Ciprofloxacin
	Third-generation cephalosporins	Ceftriaxone and ceftazidime
	Carbapenems *	Meropenem

**Key:****^‡^** The listed antimicrobials are priorities for surveillance of resistance in each pathogen, although they may not be first-line options for treatment. One or more of the drugs listed may be tested. ^a^ Oxacillin is a surrogate for testing reduced susceptibility or resistance to penicillin; the AST report to clinicians should state reduced susceptibility or resistance to penicillin. ^b^ Cefoxitin is a surrogate for testing susceptibility to oxacillin (methicillin, nafcillin); the AST report to clinicians should state susceptibility or resistance to oxacillin. ^c^ Minimum inhibitory concentration (MIC). * Imipenem or meropenem is preferred to represent the group when available.
